# Preliminary Insights into the Cyto/Genoprotective Properties of Propolis and Its Constituent Galangin In Vitro

**DOI:** 10.3390/toxics13030194

**Published:** 2025-03-08

**Authors:** Mateo Jakac, Irena Brčić Karačonji, Andreja Jurič, Dražen Lušić, Danijel Milinčić, Aleksandra Dramićanin, Mirjana Pešić, Nediljko Landeka, Nevenka Kopjar

**Affiliations:** 1 Department of Epidemiology, Teaching Institute of Public Health of Istria County, 52000 Pula, Croatia; mateo.jakac@gmail.com (M.J.); 3kazuna@gmail.com (N.L.); 2 Division of Toxicology, Institute for Medical Research and Occupational Health, 10000 Zagreb, Croatia; ajuric@imi.hr (A.J.); nkopjar@imi.hr (N.K.); 3 Department of Basic Medical Sciences, Faculty of Health Studies, University of Rijeka, 51000 Rijeka, Croatia; 4 Department of Health Ecology, Faculty of Medicine, University of Rijeka, 51000 Rijeka, Croatia; 5 Department of Environmental Health, Teaching Institute of Public Health of Primorje-Gorski Kotar County, 51000 Rijeka, Croatia; 6 Department of Chemistry and Biochemistry, Faculty of Agriculture, University of Belgrade, 11080 Belgrade, Serbia; milincic93@gmail.com (D.M.); mpesic@agrif.bg.ac.rs (M.P.); 7 Department of Analytical Chemistry, Faculty of Chemistry, University of Belgrade, 11158 Belgrade, Serbia; akosovic@chem.bg.ac.rs

**Keywords:** propolis, galangin, phenolics, irinotecan, cytokinesis-block micronucleus cytome assay, lymphocytes

## Abstract

Propolis has been well known for centuries as a natural preventive and therapeutic agent. Its numerous health benefits are mainly attributed to its high content of phenolic compounds that have a remarkable antioxidant activity. Since phenolics may exert a dual nature (pro-oxidant and antioxidant) the aim of this study was to investigate the safety profile of the ethanolic extract of propolis and the related flavonoid galangin and their ability to protect lymphocytes from irinotecan-induced cyto/genotoxicity in vitro. Isolated human peripheral blood lymphocytes were exposed for 3 h to three concentrations of propolis extract and galangin corresponding to the average daily dose of 0.25 mL of extract [propolis in 70% ethanol (3:7, *w*/*w*)], as well as a five- and ten-fold higher concentration. Cyto- and genoprotective effects were tested using a cytokinesis-block micronucleus cytome assay. Treatment with propolis and galangin in the selected concentrations exerted high biocompatibility with lymphocytes and diminished the level of cytogenetic damage caused by irinotecan. Propolis at the same concentration offered a stronger protective effect than single galangin. Also, apoptosis was the prevailing mechanism of cell death in our experimental conditions. These preliminary results speak in favour of future investigations of propolis using other available cytogenetic methods and cell models.

## 1. Introduction

Propolis or bee glue is a natural bee product collected by honeybees, *Apis mellifera* L., from living plants, partially digested by *β*-glycosidase from their saliva and mixed with beeswax [1,2]. The primary sources of European propolis (so-called poplar type of propolis) are plants from the *Populus* spp., mostly *P. nigra* L. [3,4]. Numerous studies have proven its versatile pharmacological activities: antibacterial, antifungal, antiviral, anti-inflammatory, hepatoprotective, antioxidant, and antitumour activities. These beneficial effects are associated with bioactive compounds such as galangin, caffeic acid, chrysin, pinocembrin, naringenin, quercetin, apigenin, etc. [1,4]. Poplar-type propolis is rich in antioxidants such as flavonoids, phenolic acids, and their esters that protect cells from oxidative stress [3]. On the other hand, at higher doses or under certain conditions, the pro-oxidant properties of these compounds could contribute to cyto/genotoxicity [5].

The safety and biocompatibility of propolis extract have not yet been documented well at cell level. This motivated us to explore its toxicological profile. Furthermore, since natural products are attracting great attention due to their ability to protect normal cells from cytogenetic damage during chemotherapy, we also investigated the ability of propolis extract and its constituent galangin to protect lymphocytes from irinotecan-induced cyto/genotoxicity in vitro.

There is a generally accepted notion that poorly characterised bioactive compounds and their complex mixtures, especially those contained in nutritional or natural medicinal products, must be confirmed as safe for consumers. The UK Committee on Mutagenicity of Chemicals in Food, Consumer Products and the Environment proposed a very comprehensive guidance on the strategy for genotoxicity testing of chemicals [6]. The available in vitro assays are generally classified (i) based on genetic endpoints, namely gene mutation, clastogenicity, aneugenicity and tests for DNA damage; or (ii) based on different phylogenetic levels, namely bacteria, and mammalian cells. Studies like ours belong to Stage 1 of testing, where a combination of two in vitro genotoxicity tests is usually used: the bacterial Ames test complemented with the in vitro micronucleus test. Both tests have been approved by the Organisation for Economic Co-operation and Development—OECD—in the Test Guidelines Nos. 471 [7] and 487 [8]. The rationale for such a testing strategy is to obtain information on gene mutation, changes to chromosome structure and changes to chromosome number. If further characterisation of the tested compound is needed, among the available assays there is also the in vitro mammalian chromosomal aberration test (OECD Test Guideline No. 473) [9], focused on the identification of substances that cause structural chromosomal aberrations in cultured mammalian cells. Another useful in vitro method for the estimation of genotoxicity is a versatile alkaline comet assay, which enables specific detection of DNA strand breaks, apurinic/apyrimidinic sites, modified bases, crosslinks and other features like pyrimidine dimers and DNA adducts [10].

For this study, we selected an in vitro approach using a human lymphocyte model. Our recent in vitro studies with the same cell model [11,12] demonstrated that strawberry tree (*Arbutus unedo* L.) honey, also rich in phenolic content, and homogentisic acid, a main phenolic constituent of strawberry tree honey, showed high biocompatibility at the cell level as well as remarkable geno- and cytoprotective properties when administered with the antineoplastic drug irinotecan (IRI) used to treat metastatic colorectal cancer and different solid tumours [13].

Based on encouraging results from our previous studies on honey, we applied the same experimental model on propolis and its phenolic constituent galangin in order to investigate their safety profile and their ability to protect lymphocytes from IRI-induced cytotoxicity in vitro. Among the flavonoids found in propolis, we chose galangin for the study since, according to previous research, it is considered one of the main bioactive components in the majority of Croatian propolis samples [14,15,16,17].

In order to test the cytogenetic effects of propolis and galangin, we used a cytokinesis-block micronucleus (CBMN) cytome assay on peripheral blood lymphocytes, one of the most widely applied methods in toxicology [18].

We expected this study to indicate directions for further experiments on propolis and its phenolic constituents using other available cytogenetic methods and cell models.

## 2. Materials and Methods

### 2.1. Chemical and Reagents

Galangin and naringenin were purchased from LGC Standards (Wesel, Germany), while gallic acid, Folin–Ciocalteu’s reagent, penicillin, streptomycin, cytochalasin B and acetic acid 99.5% were obtained from Sigma-Aldrich (Steinheim, Germany). Aluminium chloride, potassium chloride, potassium hydroxide, sodium carbonate, 2,4-dinitrophenylhydrazine 97% (DNP), acetic acid 99.5%, sulphuric acid 96% and ethanol 96% were products of Kemika (Zagreb, Croatia), while 2,2-diphenyl-1-picrylhydrazyl (DPPH) and trolox (6-hydroxy-2,5,7,8-tetramethylchroman-2-carboxylic acid) were obtained by Fluka (Buchs, Switzerland). Acetonitrile (MS grade), methanol (HPLC grade), formic acid (LC-MS grade) and Giemsa stain were purchased from Merck (Darmstadt, Germany). Irinotecan (IRI), in the form of hydrochloride trihydrate salt, was purchased from LC Laboratories (Woburn, MA, USA). Bleomycin, in the form of bleomycin sulphate, was the product of Nippon Kayaku Co., Ltd., Tokyo, Japan, and 0.9% sodium chloride solution was the product of the Croatian Institute for Transfusion Medicine (Zagreb, Croatia). Inactivated foetal calf serum and RPMI-1640 medium were obtained from Gibco (Grand Island, NY, USA). Phytohemagglutinin was purchased from Remel (Lenexa, KS, USA).

Ultrapure water was produced using a Direct-Q^®^ 5 UV Water Purification System (Millipore SAS, Molsheim, France).

### 2.2. Instrumentation

For spectrophotometric assays, absorbance was measured using a Cary 60 UV-Vis spectrometer (Varian Inc., Palo Alto, CA, USA). The analyses of galangin were carried out on an Agilent 1290 Infinity ultra-high-performance liquid chromatography (UHPLC) system coupled with 6530C quadrupole time-of-flight mass spectrometry (Q-ToF-MS) from Agilent Technologies, Inc. (Santa Clara, CA, USA). Cultures were maintained in the incubator (Heraeus Hera Cell 240, Langenselbold, Germany). Slides were analysed using a light microscope (Leitz, Oberkochen, Germany).

### 2.3. Propolis Sampling

**Figure 1:**
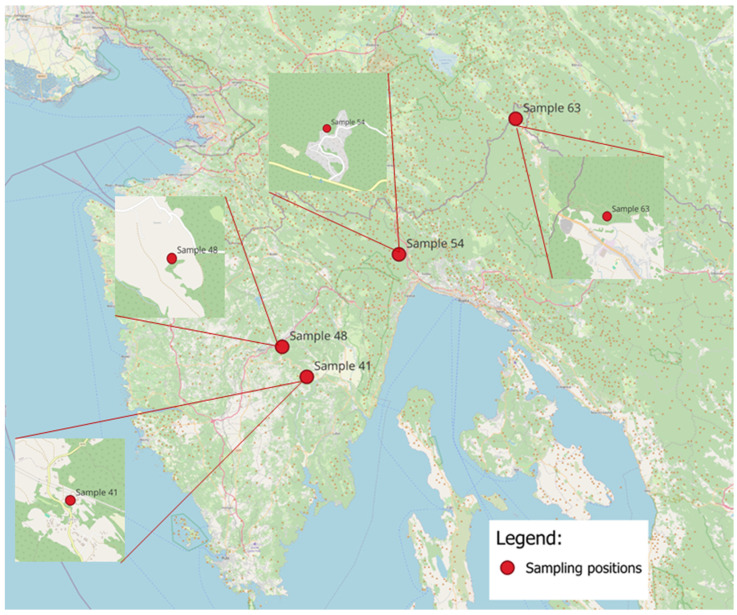
Sampling locations in Istria and Primorje-Gorski Kotar County, Croatia.

Raw propolis was collected from four locations (Figure 1): Kukurini (sample 41; 45°11′25″ N 14°03′01″ E) and Šestani (sample 48, 45°14′40″ N 13°59′16″ E) in Istria County; and Zaluki (sample 54, 45°24′25″ N 14°16′47″ E) and Prezid (sample 63, 45°38′38″ N 14°34′22″ E) in Primorje-Gorski Kotar County, Croatia. Samples were chosen according to a screening of 200+ propolis samples collected at 60 locations, singling out those that showed the highest level of total flavonoids.

Propolis samples were collected using a special silicone net that has a certificate of inertness to avoid any chemical or physical contamination. Nets with collected raw propolis samples were put into the freezer at −20 °C to facilitate the removal of raw propolis from the net, which was then stored at −20 °C. Prior to extraction, frozen propolis was homogenised by grounding in ceramic mortar. Propolis extract was prepared according to instructions from beekeepers: grounded propolis was mixed with 70% ethanol (3:7 *w*/*w*) and kept for 40 days at room temperature in amber glass bottles. Suspension was briefly mixed every day to improve extraction. After 30 days, the suspension was transferred in ceramic mortar, additionally homogenised and transferred back to the bottle for 10 more days, when the suspension was filtered using filter paper. Ethanolic extract was stored in glass amber bottles at +4 °C until analysis.

### 2.4. Determination of Total Phenolic Content

Determination of total phenolic content (TPC) was performed according to the procedure described by Jurič et al. [19], with minor modifications. Ethanolic propolis extract was diluted (1:100) with 70% ethanol (*v*/*v*) and 100 µL of diluted extract was mixed with 100 µL of 2 mol/L Folin–Ciocalteu’s reagent and 1.4 mL of ultrapure water. After 5 min, 1.5 mL of 6% (*w*/*v*) sodium carbonate solution was added and the obtained solution was incubated at 40 °C for 30 min. Absorbance was measured at 760 nm. The total phenolics were quantified from a standard curve of gallic acid solution (10–500 mg/L) and results were expressed as mg gallic acid equivalents (GAE) per g of raw propolis.

### 2.5. Determination of Total Flavonoid Content

#### 2.5.1. Flavones/Flavonols

Determination of flavones and flavonols was performed as described by Trusheva et al. [20], with minor modifications. Ethanolic propolis extract was firstly diluted (1:100) with 70% ethanol (*v*/*v*) and 400 µL of diluted extract was mixed with 700 µL of methanol and 100 μL of 5% aluminium chloride solution in methanol (*w*/*v*). After incubation at room temperature for 30 min in the dark, absorbance was measured at 425 nm. Total flavone/flavonol content was determined from a standard curve of galangin (5–250 mg/L) and results were expressed as mg galangin equivalents (GE) per g of raw propolis.

#### 2.5.2. Flavanones/Dihydroflavonols

Total flavanone and dihydroflavonol content was determined following a modified protocol, as described previously [20]. Briefly, 100 µL of ethanolic propolis extract was mixed with 200 µL of DNP (1 g of DNP was dissolved in 2 mL of 96% sulphuric acid and diluted to 100 mL with methanol). The mixture was incubated at 50 °C for 30 min. After cooling to room temperature, 700 μL of 10% potassium hydroxide in methanol (*w*/*v*) was added. An aliquot of 50 μL of reaction mixture was mixed with 2.450 mL of methanol and absorbance was measured at 486 nm. Total flavanone/dihydroflavonol content was determined from a standard curve of naringenin (1–30 g/L) and results were expressed as mg naringenin equivalents (NE) per g of raw propolis.

### 2.6. Determination of Antioxidant Activity

DPPH radical scavenging activity was measured according to the procedure described by Tuberoso et al. [21]. Ethanolic propolis extract was diluted (1:100) with 70% ethanol (*v*/*v*) and 150 µL of diluted extract was mixed with 1.85 mL of methanol and 1.5 mL of DPPH methanolic solution (0.18 mmol/L). Absorbance was measured at 517 nm after incubation in the dark at 23 °C for 30 min. The calibration curve was made using trolox (0–80 µmol/L). The results were expressed as µmol of trolox equivalents (TE) per g raw propolis.

### 2.7. Quantification of Galangin Using Ultra-High-Performance Liquid Chromatography Quadrupole Time-of-Flight Mass Spectrometry (UHPLC Q-ToF MS)

Samples of ethanolic propolis extract were diluted (1:10 *v*/*v*), with a mixture of mobile phases {[mobile phase A (water + 0.1% formic acid)]:[mobile phase B (water + 0.1% formic acid)] = 1:1}, intensively vortexed and filtered through 0.22 µm filters before analyses.

The analyses were carried out on a UHPLC Q-ToF-MS system, as previously described in detail by Kostić et al. [22]. The quantification of galangin, which is considered one of the major bioactive compounds reported in most samples of Croatian propolis [14,15,16,17], was part of a broader study on the phenolic profiling of Croatian propolis, carried out to complete the doctoral thesis of one of the authors. The QToF-MS system was equipped with a Dual Agilent Jet Stream electrospray ionisation (ESI) source, operating in both positive (ESI+) and negative (ESI−) ionisation modes. Mass spectra were recorded over the *m*/*z* range 100–1700, with a scan rate of 2 Hz. The operating parameters for ESI were the same as previously reported by Kostić et al. [22]. Agilent Mass Hunter software ver. 10.0 was used for instrument control, data acquisition and analysis. Galangin was quantified by direct comparison with available standard. Results were expressed as mg galangin per g of raw propolis.

### 2.8. Cytokinesis-Block Micronucleus (CBMN) Cytome Assay on Peripheral Blood Lymphocytes

The CBMN assay was performed in line with standard protocols [8,23].

#### 2.8.1. Blood Sampling

The peripheral blood sample used in the experiment was donated by a non-smoking 28-year-old male volunteer. The donor was not exposed to genotoxic agents or subjected to medical irradiations for one year preceding the study. The concept of the research was explained to him in detail, and his informed consent was obtained. The experimental design, personal data collection and details on blood sampling were approved by the institutional ethics board (Document Class: 01-18/23-02-2/1; Reg. No.: 100-21/22-11 issued by the Ethics Committee Institute for Medical Research and Occupational Health, Zagreb, Croatia). The total amount of blood taken from the donor by venepuncture was 50 mL. To collect the blood sample, lithium heparin-coated tubes were used (BD vacutainer^®^ with LH 170 I.U., Becton Dickinson: BD-Plymouth PL6 78P, UK).

#### 2.8.2. Lymphocyte Treatment

A comprehensive explanation of the experimental schedule is reported in Table 1. There were 15 experimental groups altogether. The same experimental design was applied in two independent trials, each of which had two replicate cultures per experimental group; therefore, four replicate cultures for every single experimental group were established.

Such an experimental design was selected due to OECD guidelines [8], which specify that: “in cytoB-treated cultures, micronucleus frequencies should be analysed in at least 2000 binucleate cells per concentration and control, equally divided among the replicates, if replicates are used”.

Control was used with solvent since the OECD protocol specifies that “Concurrent negative controls, consisting of solvent alone in the treatment medium and processed in the same way as the treatment cultures, should be included for every harvest time”. In our experiment, the tested compounds were dissolved in ethanol. We used the final concentration of the solvent as 0.03% in the culture medium, according to previous experiences in our laboratory. This was also in line with the OECD protocol, which states that organic solvents should not exceed 1% (*v*/*v*).

Bleomycin (dissolved in 0.9% sodium chloride solution) was selected as the positive control according to our earlier in vitro experiments on the same cell model [11,12].

Our decision regarding the lowest tested concentration of propolis (Propolis 1×) relied on an intake of five drops (0.25 mL) of prepared propolis extract/day by an adult person weighing 70 kg (corresponding to 1.36 mg of raw propolis/kg of body mass). To search for possible cytogenetic effects, it was proposed to evaluate 5× and 10× higher concentrations as well.

The lowest tested galangin concentration (Galangin 1×) was calculated based on its content in the average daily intake of prepared propolis extract, i.e., 1.56 mg/0.25 mL.

The IRI concentration corresponded to 350 mg/m^2^ of drug given as monotherapy to colorectal cancer patients [24]. Prior to use for the treatments, it was dissolved in 0.9% solution of sodium chloride.

Lymphocyte cultures were established as follows: aliquots of heparinised blood (V = 600 µL per culture) were pipetted into a growth medium, RPMI-1640, supplemented with 10% foetal calf serum and antibiotics (penicillin and streptomycin). To induce cell division prior to exposure to the test chemicals, lymphocytes were stimulated with the mitogen phytohemagglutinin (PHA). Cultures were kept in a humidified atmosphere of 5% CO_2_ at 37 °C.

Treatments with the test compounds were performed according to the latest OECD recommendations, given in Test Guideline No. 487—In Vitro Mammalian Cell Micronucleus Test [8]. They started 41 h after PHA stimulation and lasted for 3 h at 37 °C.

Upon completion of exposure, lymphocyte cultures were centrifuged at 100× *g*. The growth medium with the tested compounds was removed by pipetting and discarded, while the cell pellet was resuspended in a fresh amount of RPMI-1640 growth medium that contained 6 µg/mL of Cytochalasin B (cytoB). This procedure was timed for the 44th hour of lymphocyte cultivation, which represents a critical point for adding of cytoB to hinder cytokinesis and obtain binucleated cells. All treated and control cultures were further maintained at 37 °C until the end of the 72nd hour of cultivation, according to the above-mentioned standard protocols.

#### 2.8.3. Cell Harvest and Slide Preparation

The first step in the preparation of microscopic slides was harvesting of cells by centrifugation (100× *g*), followed by treatment with 0.075 mol/L potassium chloride and succeeded by centrifugation. The sediments of cells were repeatedly fixed with methanol/acetic acid (3:1 *v*/*v*) followed by centrifugation. The final lymphocyte suspension was pipetted onto clean microscope slides. For staining, 5% aqueous solution of Giemsa dye was used. Slides were screened using a light microscope at 1000× magnification. For morphological discrimination of micronuclei (MNi), nuclear buds (NBs), nucleoplasmic bridges (NPBs) and cells in apoptosis or necrosis, the criteria by Fenech et al. [25] and Fenech [23] were applied. To determine their frequencies, per experimental point, a total of 4 × 1000 binucleated (BN) cells were scored.

On the same slides, parameters of cell proliferation and cytotoxicity were also evaluated, in line with the OECD protocol [8]. For that purpose, the numbers of cells with one to four nuclei (so-called M1, M2, M3 and M4 cells) were scored using a light microscope (at 400× magnification). The total number of these cells counted per experimental group was 2000 (i.e., 4 × 500 cells).

Then, the cytokinesis-block proliferation index (CBPI) was calculated, using the following formula: CBPI = [(No. of mononucleated cells) + (2 × No. of binucleated cells) + (3 × No. of multinucleated cells)]/(Total number of cells).

The replication index (RI), which points to the relative number of cell cycles per cell during the period of exposure to cytoB in treated compared to control cultures, was computed as follows:

RI = {[(<No. binucleated cells> + <2 × No. multinucleated cells>)/(Total number of cells in treated culture)]/[(<No. binucleated cells> + <2 × No. multinucleated cells>) × (Total number of cells in control culture)]} × 100.

The calculated values of RI were used to establish cytotoxicity: % Cytotoxicity = 100 − RI.

All of the previously mentioned formulae were taken from the last OECD protocol [8], according to which data analysis, interpretation and reporting were also performed.

### 2.9. Statistics

For data analysis, the Statistica–Data Science Workbench software, version 14.0.0.15. (TIBCO Software Inc., Palo Alto, CA, USA), was used. Basic descriptive statistical parameters were determined first. Further analyses included one-way ANOVA followed by post hoc Tukey’s HSD test for multiple comparisons between groups. To find the significance of differences in results obtained for lymphocyte proliferation, Pearson’s *χ*^2^ test was used. The level of statistical significance was set at *p* < 0.05.

## 3. Results and Discussion

### 3.1. Propolis Sample Selection for Research on Biological Effects

The total phenolics, total flavonoids and galangin content as well as antioxidant activity were determined in four samples of propolis collected from western Croatia (Istria and Primorje-Gorski Kotar counties) as part of the preliminary research. The content of total phenols, flavones, flavonols, flavanones, dihydroflavonols and antioxidant capacity (DPPH assay) was determined using spectrophotometric methods.

The UHPLC Q-ToF MS technique was used to determine the level of galangin in propolis samples. Galangin content in samples ranged from undetected to 16.52 mg/g raw propolis.

A propolis sample from Istria County (sample P41) was selected for further research as it contained the highest level of total flavonoids and galangin and a similar antioxidant activity as the other samples (Table 2).

### 3.2. Cytokinesis-Block Micronucleus (CBMN) Cytome Assay

Following the initial chemical characterisation of the selected propolis samples that revealed the one with the highest content of galangin, we further investigated its potential cyto/genoprotective effectiveness using human peripheral blood lymphocytes as a model system. As an appropriate initial test for the screening and assessment of biocompatibility, the CBMN assay was selected, considering that this multi-endpoint cytogenetic technique represents a comprehensive system for the simultaneous detection of DNA/chromosome damage, cytostasis and cytotoxicity [18,23].

Our research is the first to document the biological and pharmacological activity of this type of propolis sample, since it has not been previously used in similar experiments in vitro. It is no surprise, then, that the existing knowledge regarding the biological effects of the different types of propolis studied so far on the same experimental model is quite limited.

Among the most relevant previous studies on a human lymphocyte model, there have been two reports from Turkey [26,27]. In the first study [27], the CBMN assay was used and the authors concluded that incubation of cells with 0.01, 0.05, 0.1, 0.2, 0.5, 0.7 and 1.0 mL of propolis did not result in a significant detrimental effect to lymphocytes, although there was a concentration-dependent tendency towards increasing MNi rates. Furthermore, higher concentrations of propolis also significantly lowered mitotic index rates compared to control. In their second study [26], another cytogenetic endpoint, sister chromatid exchanges (SCEs), was studied, and lymphocytes were exposed for 70 h to 5, 25, 50 and 250 mg/mL of propolis. Increased SCE rates indicated that the tested propolis produced genotoxic effects at high concentrations.

Benković et al. [28,29] reported the first results regarding the effectiveness of propolis sampled in Croatia in reducing cytogenetic damage in human peripheral lymphocytes induced by ionising radiation. Both studies used the alkaline comet assay to measure the levels of primary DNA damage, while cytogenetic damage was assessed by structural chromosome aberration analysis and CBMN assay. It has to be stressed that the authors tested propolis collected from beehives kept on the outskirts of Zagreb, Croatia, which is rather distant from the region where our propolis sample was collected. It is well known that the chemical composition of propolis depends greatly on geographical and climatic factors, plant resources and collecting season [4]. Further, their studies were focused on two different extracts: ethanolic propolis extract and the water-soluble derivative of propolis. As individual compounds whose effects were evaluated, they selected quercetin, caffeic acid, chrysin and naringin. The findings reported in the above-mentioned papers showed an acceptable toxicity profile of both propolis extracts and their individual constituents, and confirmed their radioprotective abilities. However, since Benković et al. [28,29] did not investigate the effects of galangin, our study provides the first evidence regarding galangin biocompatibility when tested at levels comparable to those measured in the selected Croatian propolis sample.

With regard to earlier cytogenetic studies with propolis, we should also mention those conducted in Spain by Montoro et al. [30,31]. The first [30] focused on investigating the radioprotective effect of propolis against chromosomal damage induced in lymphocytes exposed to 2 Gy γ-rays. The obtained results confirmed that pre-treatment with the ethanolic extract of propolis (EEP) led to a significant and concentration-dependent decline in the frequency of chromosome aberrations. The results of the second study [31] showed that high concentrations of EEP (500 to 2000 μg/mL) could have a cyto- and genotoxic effect, since they significantly lowered the mitotic index and proliferation index in lymphocyte cultures and increased SCE rates.

In contrast to propolis, there are very little data available on the effect of galangin on genome stability in human lymphocytes. Bacanlı et al. [32] studied the antioxidant, cytotoxic and antigenotoxic effects of galangin (at 10, 100, 500, 1000, 2000, 5000, 10,000 and 20,000 μmol/L) using CBMN and alkaline comet assays. They found that galangin was not genotoxic per se but efficiently reduced the frequency of MNi and DNA damage induced by hydrogen peroxide in lymphocytes.

With the aim of clarifying previously unexplored biological effects of the selected propolis sample, and especially the poorly documented effects of galangin, in more detail, this study applied a similar experimental design as reported previously in two trials by Jurič et al. [11,12]. These studies investigated the protective effects offered by strawberry tree honey and its dominant compound, homogentisic acid, when applied in combination with the cytotoxic drug IRI. The advantage of this approach is a simple experimental setup, speed of performance, robustness of the used CBMN test system and high reproducibility of the obtained results.

IRI was intentionally selected as a producer of genome instability in this experiment since, nowadays, it represents one of the most important drugs used in cancer treatment. Its efficacy is continuously improved, using new pharmaceutical technologies as well [33]. Although its main target is the DNA topoisomerase I enzyme, IRI acts through various mechanisms of action, which have been extensively reviewed in several recent papers [34,35,36,37].

No less important, the detrimental effects of IRI on a human lymphocyte model were extensively studied and documented in our laboratory earlier, using a battery of endpoints [38]. It was observed that at the same concentration as used in the present study, IRI was a strong inducer of apoptosis and necrosis in lymphocytes; it significantly increased the levels of primary DNA damage and cytogenetic damage (especially chromatid breaks and complex quadriradials, but also translocations, involving chromosomes 1, 2 and 4). Treatment also led to increased frequencies of MNi, NBs and NPBs, as well as to an increased rate of SCE, while lymphocyte proliferation was markedly decreased.

Taking all of the above into account, we started from the following premise: if we are familiar with the extent and types of cytogenetic damage that IRI causes, this could help us to estimate more accurately the level of probable protective potential that the tested propolis samples, or galangin, can provide.

Table 3 and Table 4 report the results of the CBMN assay, along with detailed intergroup differences and their statistical significances. In the control group, a low level of cytogenetic damage, comparable to historical controls in the laboratory [39], was observed. Treatment with propolis extract and galangin in the selected concentrations did not cause a significant increase in the total amount of MNi. Most of BN cells in control, propolis- and galangin-treated samples contained one MN. Treatments also did not stimulate a significant formation of NBs. For single propolis, we noticed a slight concentration–response relationship for NB formation. However, most of the BN cells in control, propolis- and galangin-treated samples contained one NB. In those experimental groups, no formation of NPBs was noticed. Such results suggest that propolis extract and galangin at the tested concentrations could be considered biocompatible.

Binucleated cells with more MNi or NBs were observed in IRI-treated samples and in the positive control (bleomycin). In the latter experimental group, as expected, the highest level of MNi, NBs and NPBs was observed. Treatment with IRI resulted in an increased incidence of all of the aforementioned descriptors of the CBMN assay. Observations regarding IRI-induced genome instability were in line with previous reports obtained on a lymphocyte model [11,12,38].

Propolis extract and galangin at the tested concentrations diminished the level of cytogenetic damage caused by IRI, but with different potency (Table 3 and Table 4).

The mechanisms and processes behind the formation of nuclear anomalies detected by the CBMN assay are well known [18] and comprise the following: (1) structural/numerical chromosome aberrations and spindle/kinetochore defects leading to chromosome mis-segregation during mitosis (expressed as MNi), (2) the formation of anaphase bridges (expressed as NPBs) and (3) gene amplification or elimination of unresolved DNA complexes (expressed as NBs).

The decreased levels of these nuclear anomalies observed after the combined exposure of lymphocytes to IRI and propolis extract suggest that in the presence of propolis injured cells were able to activate multiple mechanisms to counteract IRI-induced lesions in DNA/chromosomes, as well as deficiencies in the components or functionality of the mitotic apparatus. The evidence regarding the higher protective potency of propolis compared to single galangin speaks in favour of a previously documented feature of propolis from other geographical regions [40] and various other complex natural products [11]. In fact, no single compound, but rather a combination of different bioactive constituents, is usually responsible for the beneficial effects observed after treatment with complex mixtures.

**Figure 2:**
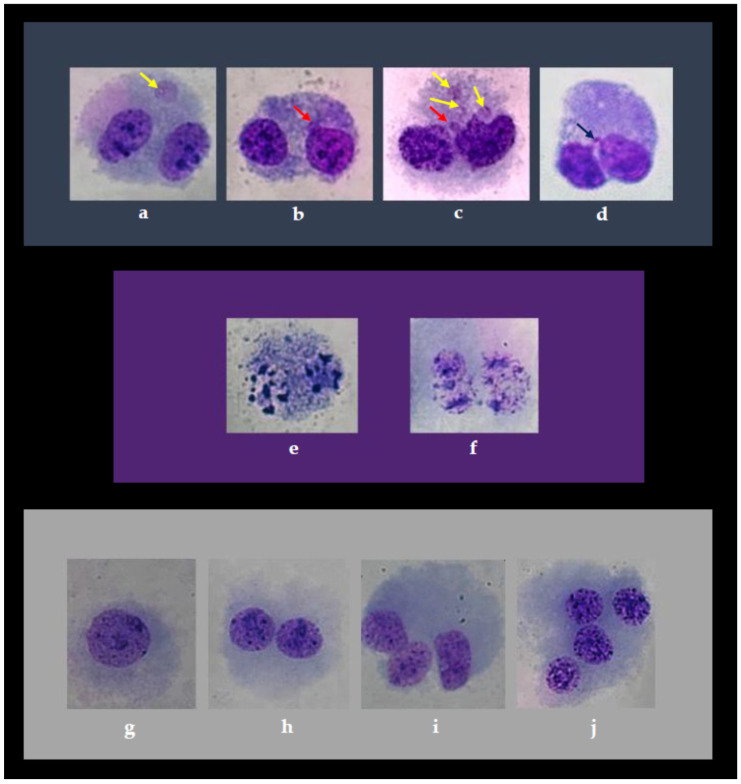
(**a**–**j**) Typical features of human peripheral blood lymphocytes visible on microscope slides stained with a Giemsa dye, prepared using the methodology of the CBMN cytome assay. Individual photomicrographs in the first row show (**a**) a binucleated lymphocyte with a micronucleus, MN (marked with a yellow arrow), in the galangin 1× + IRI experimental group; (**b**) a binucleated lymphocyte with a nuclear bud, NB (marked with a red arrow), in the IRI experimental group; (**c**) several MNi (marked with yellow arrows) and an NB (marked with a red arrow) are visible in a lymphocyte following treatment with single IRI; (**d**) two nuclei connected by a nucleoplasmic bridge, NPB (marked with a black arrow), following treatment with single IRI. Individual photomicrographs in the second row show morphological features of dead cells following treatment with single IRI: (**e**) an apoptotic cell with nuclear fragmentation; (**f**) a necrotic cell. Individual photomicrographs in the third row show typical features of cells scored to determine the cytokinesis-block proliferation index: (**g**) mononucleated cell, M1, in the galangin 10× + IRI experimental group; (**h**) a cell with two nuclei, M2, in the galangin 10× + IRI experimental group; (**i**) a cell with three nuclei, M3, in the IRI experimental group; (**j**) a cell with four nuclei, M4, in the galangin 10× + IRI experimental group. Photographed at magnification ×1000 with Axiocam 208 colour camera on Axiolab 5 microscope (Carl Zeiss Microscopy GmbH, Jena, Germany).

The typical appearance of binucleated cells with cytogenetic damage as observed on microscope slides is shown in Figure 2.

The extent of protective effect offered by propolis and galangin against IRI-induced cytogenetic damage was confirmed by further calculations of the mutagenicity index (MutI), based on the formula proposed in the paper of Scarpato et al. [41], which was also successfully applied in our previous publications [11,12].

**Figure 3:**
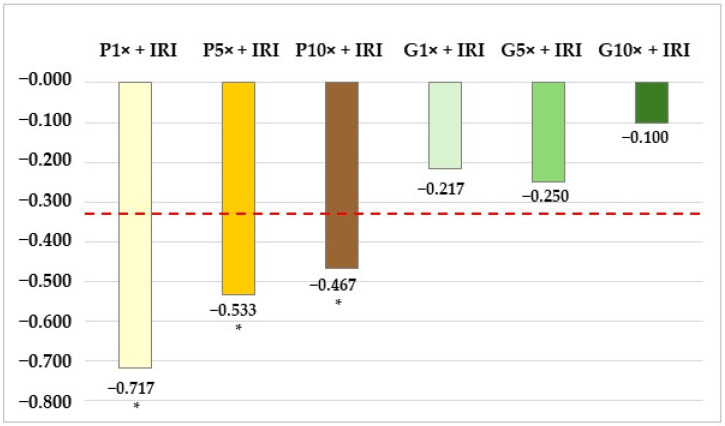
Influence of propolis (P) and galangin (G), both tested at 1×, 5× and 10× concentrations, on the reduction in cyto/genotoxic effects caused by irinotecan (IRI). Columns represent the values of the mutagenicity index (MutI), determined using the formula proposed in the paper of Scarpato et al. [41]. The dashed red line represents the calculated cut-off value of MutI (−0.333) that denotes an MNi frequency decrease significant at a 5% significance level. All MutI values marked with the symbol * are considered significantly lower compared to the cut-off value.

The starting point for these calculations was the total number of MNi determined in the sample treated with IRI (60 MNi per 4000 binucleated cells). This value was first used to estimate the number of MNi required to reach a decrease and an increase considered significant at a 5% significance level. The resulting values were 40 MNi and 84 MNi, respectively. These numbers were further used to calculate cut-off values of MutI, which were −0.333 for the MNi frequency decrease and 0.400 for the MNi frequency increase. When the total numbers of MNi scored in samples exposed to single IRI (60 MNi) and in all combined treatments (i.e., propolis with IRI and galangin with IRI) were entered into the formula, we obtained the resulting values of the MutI as shown in Figure 3. Since the interpretation proposes that the absolute value of the obtained index must be higher than the absolute value corresponding to the cut-off, the data obtained in this experiment confirmed a marked cytoprotective potential of propolis at all three of the tested concentrations. Interestingly, galangin at the present exposure scenario offered much lower cytoprotection against IRI-induced damage.

Although data regarding MutI values obtained in different studies cannot be simply inter-compared, primarily because lymphocytes originate from different donors and due to specific exposure conditions, it is indeed interesting to mention some trends in the results. Our present findings indicate that the protective potential of propolis (at the concentration equal to 1× the daily consumed dose) against genome instability produced by the same concentration of IRI as used here was higher compared to 1× strawberry tree honey or 1× homogentisic acid, tested in the studies by Jurič et al. [11,12]. Such a result is promising and indicates that, owing to its specific chemical composition, the tested propolis sample could have potential application as a valuable natural health product. However, before drawing any specific conclusions in that regard, further research is needed.

While screening microscope preparations to determine trends in the levels of the main CBMN assay descriptors, nonviable cells were also simultaneously counted on the same slides. Their discrimination was based on the specific morphological features typical for apoptosis or necrosis. It is well known that the biological significance of these phenomena differs deeply, and that the determination of the predominant type of cell death represents a very important issue in each experimental setting.

As defined in the scoring criteria for the CBMN assay [23], apoptotic cells showed chromatin condensation within the nucleus (in the early phase) and nuclear fragmentation into smaller nuclear bodies (in late phase). Such cells also displayed greater staining intensity of the nucleus, nuclear fragments and cytoplasm compared to viable cells.

**Figure 4:**
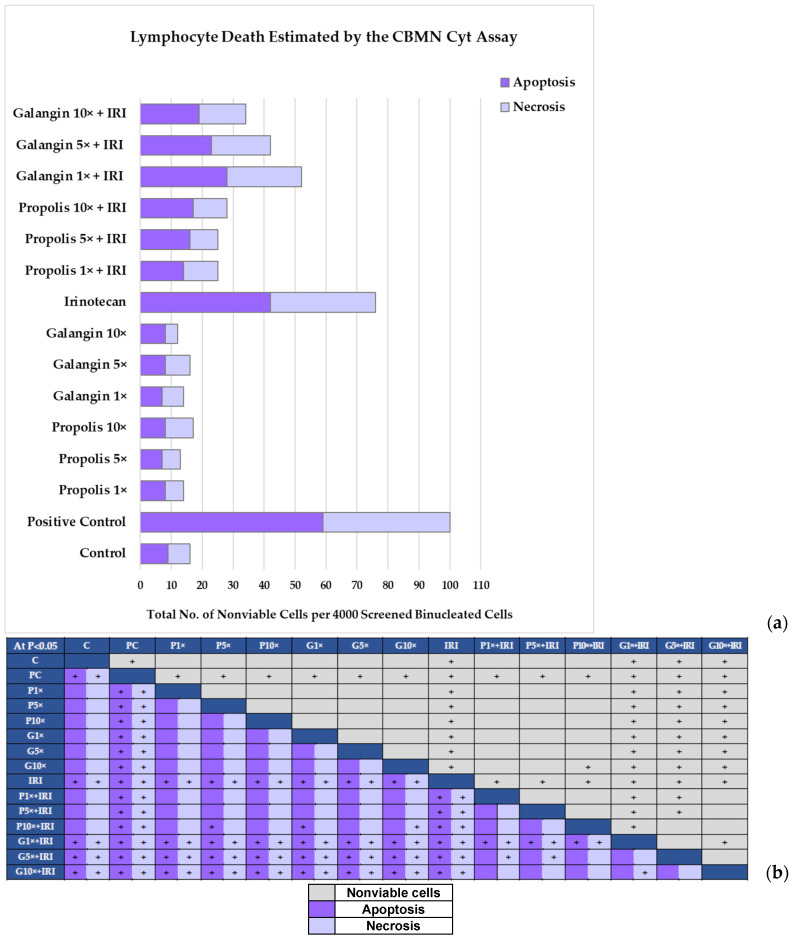
(**a**) Results regarding incidence of nonviable cells in human lymphocytes. P—exposure to propolis at 1×, 5× and 10× concentrations; G—exposure to galangin at 1×, 5× and 10× concentrations; IRI—exposure to IRI; P1× + IRI, P5× + IRI and P10× + IRI—exposure to combinations of propolis and IRI; G1× + IRI, G5× + IRI and G10× + IRI—exposure to combinations of galangin and IRI. Control and positive control (bleomycin) groups were studied in parallel. Microscopic evaluation was performed using a light microscope at 1000× magnification. Four independent scorings of binucleated cells were performed (1000 per replicate) and the total number of nonviable cells was recorded. Discrimination of nonviable cells into apoptosis or necrosis was based on their morphological features. Data are expressed as total number of nonviable cells scored along with 4000 binucleated cells for each experimental group. (**b**) Intergroup differences and their statistical significances obtained by ANOVA with post hoc Tukey’s HSD test. The left part of the table refers to results regarding numbers of cells in apoptosis and necrosis. The right part of the table refers to results regarding total number of nonviable cells. The “+” symbol indicates intergroup differences that were statistically significant at *p* < 0.05.

A major hallmark of early necrosis was a pale cytoplasm with numerous vacuoles, while the later stage was characterised with a marked loss of cytoplasm and leakage of nuclear material from the nuclear boundary. The staining intensity of the nucleus and cytoplasm in necrotic cells was lower than in viable cells. The specific features of apoptotic and necrotic lymphocytes found on microscopic slides are displayed in Figure 4.

As shown in Figure 4, treatment with propolis and galangin in the selected concentrations did not result in a significant death rate of lymphocytes. As expected, the highest number of nonviable cells was scored in the positive control and after treatment with single IRI. Propolis and galangin contributed to the reduction in lymphocyte death when administered together with IRI. Figure 4 displays the obtained results regarding the incidence of apoptosis and necrosis in all of the experimental groups (a), along with detailed intergroup differences and their statistical significances (b).

For each experimental group, we also calculated the ratio between the recorded number of nonviable cells and the cells in apoptosis (Table 5). According to reference [42], a ratio ≤ 1 means a prevalence of apoptotic events that induce cell death, while a ratio > 1 indicates the acute toxicity of the tested substance that promotes necrosis. As all obtained values were below 1, this represents further evidence that apoptosis was the prevailing mechanism of cell death in our experimental conditions.

The obtained results are important from the toxicological point of view, especially those observed after the exposure of cells to single propolis and single galangin in their three selected concentrations. The fact that apoptosis prevailed indicates the elimination of dead cells via a controlled and programmed mechanism, without producing harm to the adjacent cells, which typically occur during necrosis.

Furthermore, as apoptosis also dominated after combined exposure with IRI, such a finding suggests that both propolis and galangin help to alleviate the harm caused by mechanisms contributing to inflammatory responses associated with necrosis. Again, propolis at the same tested concentration offered a stronger protective effect than single galangin.

As shown in Figure 4 and Table 5, a slight concentration–response relationship for the total number of cells in apoptosis was also observed with increasing concentrations of propolis when applied together with IRI in the tests. This may point to the proapoptotic potency of some compounds in propolis, which is a complex mixture. However, this issue has to be clarified by more sensitive techniques in future studies

Lorge et al. [43] emphasised that the overall cytotoxicity of the tested substance in a cell culture results in both cytostasis and cell death. Although cytostasis results from the action of cell division inhibitors, it might also be related to various cytotoxicity pathways causing a delayed cell cycle. This is why the further in-depth study of the cytotoxic effects produced after lymphocyte exposure to the tested compounds involved estimations of cell proliferation parameters, including cytostasis. Here, again, the recommendations given by OECD [8] were followed.

As is known, changes in cell proliferation parameters in treated cultures compared with appropriate controls can also be considered “surrogate” estimates for cytotoxicity.

### 3.3. Analysis of Lymphocyte Proliferation

The analysis of lymphocyte proliferation in this experiment was based on the scoring of cells with one nucleus (M1), two nuclei (M2), three nuclei (M3) and four nuclei (M4), whose typical features observed on the microscope slides are shown in Figure 2.

More numeric details related to lymphocyte proliferation are reported in Table 3. Lymphocyte proliferation was highly disturbed in the positive control, where most of the cells were mononucleated and binucleated. Since that sample was treated with the cytotoxic drug bleomycin, such a result was expected.

Treatment with propolis and galangin in the selected concentrations caused slight changes in lymphocyte proliferation. Significant deviations in the percentages of M1 to M4 cells relating to the control sample are marked with arrows in Table 6.

It must be emphasised again that CBPI calculations rely on the number of nuclei per cell. In that view, the higher proportion of mononucleates included in the calculation results in lower CBPI values, which implies stronger cytostasis.

As reported in Table 6, treatment with IRI led to a marked increase in M1 cells and decreases in the percentages of M2, M3 and M4 cells, which resulted in the lowest CBPI value (1.609).

**Figure 5:**
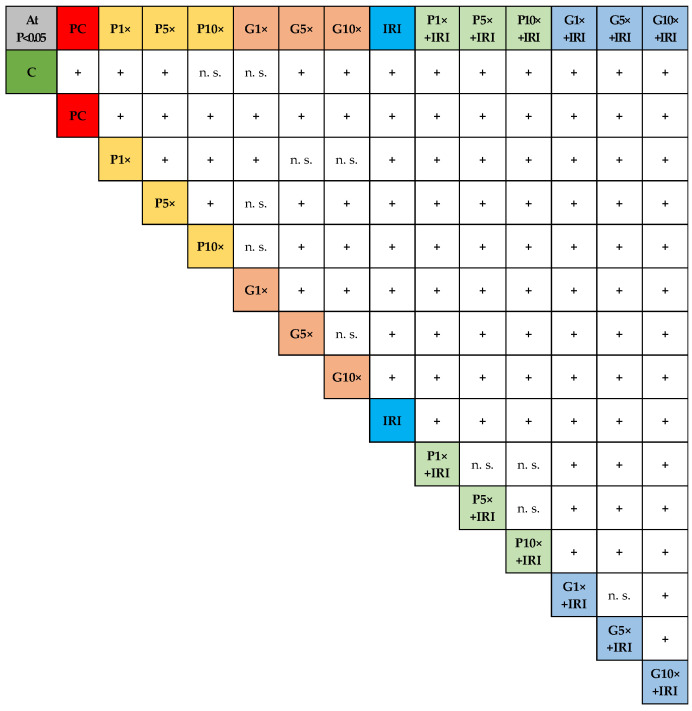
Intergroup differences regarding values of CBPI and their statistical significances obtained by Pearson’s *χ*^2^ test. Symbol “+” indicates intergroup differences that were statistically significant at *p* < 0.05; n. s.—not significant.

Detailed intergroup differences regarding all CBPI values and their statistical significances obtained by Pearson’s *χ*^2^ test are displayed in Figure 5.

Propolis and galangin, when administered together with IRI, effectively restored values of CBPI towards the value recorded in the control lymphocyte culture. Both produced significantly decreased percentages of M1 cells (marked with L in Table 6), and significantly increased percentages of M2 cells compared to the single IRI-treated sample (marked with H in Table 6). Further, in propolis + IRI cultures we also observed significantly increased percentages of M3 and M4 cells compared to single IRI-treated samples (marked with H in Table 6). Such results indicated that lymphocyte proliferation disturbed by IRI was normalised in the presence of propolis.

As another estimate of cytotoxicity, we also studied the replication index (RI). It indicates the relative number of cell cycles per cell during the period of exposure to cytochalasin B in treated cultures compared to control cultures [8].

The obtained values for the RI are reported in Table 6. The lowest values for the RI were determined for IRI-treated lymphocytes and in the positive control, confirming the strong cytostatic/cytotoxic effects produced by both compounds.

The RI value of 62% determined after treatment with single IRI means that, in terms of the cells that divided to form binucleated and multinucleated cells in the negative control culture, 62% of them divided in the IRI-treated culture. As indicated by the values calculated for the combined exposures (80.1–94.6%), lymphocyte proliferation was effectively restored both by propolis and galangin. When compared to a single IRI treatment, combined treatments with propolis and galangin resulted in an RI increase between 18.1% and 32.6% compared to IRI. The values of RI reported in Table 6 demonstrate that propolis at the same tested concentration offered a stronger protective effect than galangin. In any case, this is a favourable property of propolis if we consider that this study was conducted on a model of healthy cells. In this context, the obtained results are promising because they indicate that the presence of propolis and galangin in the case of concomitant exposure to a cytotoxic drug (in a real-life scenario, for example, during cancer chemotherapy) provides protection to healthy cells (that should be spared and protected from the unwanted side effects of therapy). Furthermore, the fact that galangin, when given in combination with IRI, showed lower CBPI and RI values points to its stronger antiproliferative potency. Such a feature would be useful for enhancing the effect of chemotherapeutics in the eradication of tumour cells. Nevertheless, these preliminary observations we made could be a matter for future studies with other cell models, especially those of tumour origin.

Finally, after describing the most important results of this research, we must briefly mention the mechanisms underlying the observed phenomena at the cellular level. Although in this experiment specific biochemical markers that could confirm some effects were not studied, we can indirectly draw some general conclusions. In that view, the knowledge from related previously published works could also be valuable.

As established in preceding studies with propolis and other natural products [3,11,19,44], their beneficial effects are principally governed by the high antioxidative potential of the tested substances. In the present study, we reported findings regarding the DPPH radical scavenging activity of the tested propolis, which confirmed its favourable properties.

Furthermore, the exceptional antioxidative/protective properties could also be related to a high level of specific phenolic compounds. In this case, the selected sample of propolis contained a high amount of galangin, which was confirmed in previous studies as an efficient antioxidant [32,45].

Moreover, we must emphasise that the effects of a complex natural product such as propolis certainly do not only depend on its qualitative and quantitative composition, but also on the mutual interactions between its components. One should also keep in mind that phenolic constituents quite often simultaneously show pro-oxidant and antioxidant properties, which could modify their biological response. It should also be taken into consideration that due to their ambivalent character, the effects caused by one compound may be reversed (sometimes even enhanced) by the impact of the other component(s) present in the complex mixture. Reports in the literature state that pro-oxidant behaviour mostly depends on the specific conditions (including the levels of molecular oxygen and some ions like copper and/or iron) that potentiate oxidative stress [46,47,48,49,50,51].

Here, we should also briefly refer to the observed effects of propolis and galangin on lymphocyte proliferation. Considering the fact that lymphocytes are primary cells with a stable genome, whose proliferative capacities, growth and division are well controlled (in contrast to various tumour-derived cell lines frequently used in toxicological studies), based on the results obtained in the present study it is hard to speculate on the mechanisms responsible for cell cycle delay and cytostasis observed after treatments. We simply cannot propose them due to the restraints of the applied methodological approach. Similarly, because we did not use specific methods for the detection of cell death, but rather estimated the number of nonviable cells based on their morphological features, we cannot propose the exact mechanisms underlying these events. Nevertheless, available, recent research includes many review papers that summarise the results reported in many previous studies focused on the antiproliferative properties and cytostatic and cytotoxic effects of various types of propolis and their bioactive components [52,53,54,55,56,57,58,59,60].

### 3.4. Limitations of This Study

We are aware that our study has certain limitations. An important issue is the problem with the standardisation of propolis, considering that propolis samples collected in different geographical areas may vary in their phytochemical composition, and that the content of the main phenolic components may vary over the years as well. Characterising propolis by considering the total content of bioactive compounds, in this case the phenolics in ethanolic propolis extract offer advantages over analysing individual compounds because of the synergistic effects of these compounds, which contribute to the overall biological activity of propolis [61]. Since all of these factors could modify the biological response, these issues must be further studied in more detail. There are also some limitations of the experiment with the CBMN assay. Although our treatment schedule followed current OECD recommendations [8] for short treatment without S9, in the forthcoming studies an extended treatment, involving cell exposure for 1.5–2 normal cell cycle lengths in the presence of cytochalasin B has to be performed as well. A detailed analysis of the obtained results shows that for some endpoints there were trends pointing to the concentration and effect relationship. However, as the testing was conducted without metabolic activation, we do not propose any conclusions about this form of dependence at this stage of study. More complete data in that regard could be obtained after comparison with the results obtained after all exposure scenarios are repeated with S9 on the same cell model using the same assay. Additional information could be obtained using other available methods, like a comet assay or an analysis of structural chromosome aberrations, which could be coupled with specific FISH probes as well.

We are also aware that the evidence that stems from the usage of the lymphocyte model cannot be generalised. This research is of a very preliminary character and, as we have already pointed out, it is proposed as an introduction to more complex experiments that will be directed towards solving most of the open questions.

## 4. Conclusions

Our results have shown that the selected sample of Croatian propolis contained high levels of total phenolics (11.0%, in mass fraction as gallic acid), together with a galangin content of 16.52 mg/g. The mass fraction of total flavones/flavonols, as galangin, was 1.2%, while the mass fraction of total flavanones/dihydroflavonols, as naringenin, was 2.4%. The antioxidant activity of the selected propolis sample was also high (176.89 mmol TE/g).

Using the CBMN assay, we documented that short treatment of lymphocytes with the selected sample of Croatian propolis and its major constituent galangin produced negligible cyto/genotoxic effects. The mean number of MNi per 1000 BN cells induced after exposure to single propolis did not exceed 2.00, irrespective of the tested concentrations. After exposure to single galangin, it was 1.75 for the highest, 2.00 for the lowest and 2.25 for the middle tested concentration. Neither treatment with propolis nor with galangin significantly increased the incidence of NB and NPB. Treatments with propolis and galangin in the selected concentrations did not result in a significant death rate of lymphocytes. Following these treatments, lymphocyte proliferation was not impaired to a great extent.

Propolis and galangin offer marked cytoprotective effects against damage caused by IRI. While the mean number of MNi per 1000 BN cells induced after exposure to IRI was 15.00, combined treatments with propolis resulted in the lowering of the mean number of MNi per 1000 BN cells: 4.25 (P1× + IRI) < 7.00 (P5× + IRI) < 8.00 (P10× + IRI). Single galangin showed somewhat lower protective effects in that view, and the mean number of MNi per 1000 BN cells was 11.75 (G1× + IRI) < 11.25 (G5× + IRI) < 13.50 (G10× + IRI). Both compounds were able to counteract cell division impairments caused by IRI. The replication index in the IRI-treated sample was 62.0%. After combined exposure to propolis and IRI, it was in the range 92.8% (P1× + IRI) < 93.8% (P5× + IRI) < 94.6% (P10× + IRI). When given in combination with IRI, galangin at all of the tested concentrations produced higher antiproliferative effects than propolis: 80.1% (G1× + IRI) < 80.5% (G10× + IRI) < 82.0% (G5× + IRI).

Considering that these results were obtained on one specific cell model and in view of all of the limitations, the open queries that persist should be investigated in more depth in forthcoming studies by applying other exposure scenarios and other cell models.

## Figures and Tables

**Table 1:** Experimental schedule.

Experimental Group	Description
Control	Ethanol in final concentration 0.03% in the culture medium
Positive Control	Bleomycin 15 mg/L
Treatments without cytotoxic drug	
Propolis 1×	1.36 mg/L
Propolis 5×	6.8 mg/L
Propolis 10×	13.6 mg/L
Galangin 1×	0.02 mg/L
Galangin 5×	0.11 mg/L
Galangin 10×	0.22 mg/L
Treatments with cytotoxic drug	
Irinotecan (IRI)	+9.0 mg/L
Propolis 1× + IRI	1.36 mg/L + 9.0 mg/L
Propolis 5× + IRI	6.8 mg/L + 9.0 mg/L
Propolis 10× + IRI	13.6 mg/L + 9.0 mg/L
Galangin 1× + IRI	0.02 mg/L + 9.0 mg/L
Galangin 5× + IRI	0.11 mg/L + 9.0 mg/L
Galangin 10× + IRI	0.22 mg/L + 9.0 mg/L

**Table 2:** Content of phenolic compounds and antioxidant activity of propolis extracts.

Sample	Total Phenolics (mg GAE/g)	Total Flavones/ Flavonols (mg GE/g)	Total Flavanones/ Dihydroflavonols (mg NE/g)	Antioxidant Activity (DPPH) (mmol TE/g)	Galangin Content (mg/g)
P41	109.94 ± 3.95	12.48 ± 0.56	23.83 ± 0.89	176.89 ± 0.31	16.52
P48	106.40 ± 7.11	10.06 ± 0.63	22.74 ± 0.63	174.35 ± 0.20	7.37
P54	131.11 ± 8.45	9.23 ± 0.21	22.58 ± 0.57	176.31 ± 2.17	ND
P63	101.44 ± 0.40	5.09 ± 0.23	17.21 ± 0.01	181,37 ± 1.18	ND

Results are expressed per g of raw propolis as average values ± SD of three measurements, except for galangin. GAE—gallic acid equivalent; GE—galangin equivalent; NE—naringenin equivalent; TE—trolox equivalent; ND—not detected.

**Table 3:** Micronuclei (MNi) formation in human lymphocytes after treatment with propolis (at 1×, 5× and 10× concentrations), galangin (at 1×, 5× and 10× concentrations), irinotecan (IRI) or combinations of propolis/galangin and irinotecan (IRI), and in the appropriate controls.

Experimental Group	Micronuclei (MNi)							
	Mean (MNi)_1000_ ± SD	Total (MNi)_4000_	Mean (BN_MN_)_1000_ ± SD	Total (BN_MN_)_4000_	Distribution of BN_MN_ Cells with			
					1 MN	2 MN	3 MN	4 MN
Control	1.75 ± 0.50	7	1.75 ± 0.50	7	7	0	0	0
Positive Control (Bleomycin)	24.25 ± 6.24 *	97 *	19.75 ± 3.77 *	79 *	65 *	11 iri, g10+iri	2	1
Propolis 1×	2.00 ± 0.82	8	2.00 ± 0.82	8	8	0	0	0
Propolis 5×	2.00 ± 0.00	8	2.00 ± 0.00	8	8	0	0	0
Propolis 10×	2.00 ± 0.00	8	2.00 ± 0.00	8	8	0	0	0
Galangin 1×	2.00 ± 0.82	8	2.00 ± 0.82	8	8	0	0	0
Galangin 5×	2.25 ± 0.50	9	2.25 ± 0.50	9	9	0	0	0
Galangin 10×	1.75 ± 0.50	7	1.75 ± 0.50	7	7	0	0	0
Irinotecan (IRI)	15.00 ± 2.00 c,p1,p5,p10,g1,g5,g10,p1+iri, p5+iri, p10+iri	60 c,p1,p5,p10,g1,g5, g10,p1+iri, p5+iri, p10+iri	13.00 ± 0.82 c,p1,p5,p10,g1,g5, g10,p1+iri, p5+iri, p10+iri	52 c,p1,p5,p10,g1,g5,g10, p1+iri, p5+iri, p10+iri	45	6	1	0
Propolis 1× + IRI	4.25 ± 0.50	17	4.00	16	15	1	0	0
Propolis 5× + IRI	7.00 ± 1.41 c,p1,p5,p10,g1,g5,g10	28 c,p1,p5,p10,g1, g5,g10	6.75 ± 1.71 c,p1,p5,p10,g1,g5,g10	27 c,p1,p5,p10,g1,g5,g10	26 c,p1,p5, p10,g1,g5, g10	1	0	0
Propolis 10× + IRI	8.00 ± 1.41 c,p1,p5,p10,g1,g5,g10	32 c,p1,p5,p10,g1, g5,g10	7.25 ± 0.96 c,p1,p5,p10,g1,g5,g10	29 c,p1,p5,p10,g1,g5,g10	26 c,p1,p5, p10,g1,g5, g10	3	0	0
Galangin 1× + IRI	11.75 ± 4.11 c,p1,p5,p10,g1,g5,g10, p1+iri	47 c,p1,p5,p10,g1,g5, g10,p1+iri	11.50 ± 3.70 c,p1,p5,p10,g1,g5, g10,p1+iri, p5+iri, p10+iri	46 c,p1,p5,p10,g1,g5,g10, p1+iri, p5+iri, p10+iri	45 c,p1,p5,p10,g1,g5,g10,p1+iri, p5+iri, p10+iri	1	0	0
Galangin 5× + IRI	11.25 ± 0.96 c,p1,p5,p10,g1,g5,g10, p1+iri	45 c,p1,p5,p10,g1,g5, g10,p1+iri	11.00 ± 0.82 c,p1,p5,p10,g1,g5, g10,p1+iri, p5+iri	44 c,p1,p5,p10,g1,g5,g10, p1+iri, p5+iri	43 c,p1,p5, p10,g1,g5, g10,p1+iri, p5+iri, p10+iri	1	0	0
Galangin 10× + IRI	13.50 ± 1.29 c,p1,p5,p10,g1,g5,g10, p1+iri, p5+iri, p10+iri	54 c,p1,p5,p10,g1,g5, g10,p1+iri, p5+iri, p10+iri	12.25 ± 0.82 c,p1,p5,p10,g1,g5, g10,p1+iri, p5+iri, p10+iri	49 c,p1,p5,p10,g1,g5,g10, p1+iri, p5+iri, p10+iri	44 c,p1,p5, p10,g1,g5, g10,p1+iri, p5+iri, p10+iri	5	0	0

Microscopic evaluation was performed using a light microscope at 1000× magnification. To establish the frequencies of MNi, 4000 binucleated cells altogether that contained MNi (i.e., BNMN cells) were scored (1000 per replicate). Data are expressed as mean ± SD of four independent evaluations, per experimental group, and total values. The statistical significance of the results was evaluated using ANOVA with post hoc Tukey’s HSD test. The level of statistical significance was set at *p* < 0.05. The symbols and small letters indicate from which groups the relevant group differs with statistical significance: *—vs. all other experimental groups; c—vs. control; p1—vs. propolis 1× group; p5—vs. propolis 5× group; p10—vs. propolis 10× group; g1—vs. galangin 1× group; g5—vs. galangin 5× group; g10—vs. galangin 10× group; iri—vs. IRI group; p1+iri—vs. combination of propolis 1× and IRI group; p5+iri—vs. combination of propolis 5× and IRI group; p10+iri—vs. combination of propolis 10× and IRI group; g1+iri—vs. combination of galangin 1× and IRI group; g5+iri—vs. combination of galangin 5× and IRI group; g10+iri—vs. combination of galangin 10× and IRI group.

**Table 4:** Formation of nuclear buds (NBs) and nucleoplasmic bridges (NPBs) in human lymphocytes after treatment with propolis (at 1×, 5× and 10× concentrations), galangin (at 1×, 5× and 10× concentrations), irinotecan (IRI) or combinations of propolis/galangin and IRI, and in the appropriate controls.

Experimental Group	Nuclear Buds (NBs)						Nucleoplasmic Bridges (NPBs)					
	Mean (NBs)_1000_ ± SD	Total (NBs)_4000_	Mean (BN_NB_)_1000_ ± SD	Total (BN_NB_)_4000_	Distribution of BN_NB_ Cells with		Mean (NPBs)_1000_ ± SD	Total (NPBs)_4000_	Mean (BN_NPB_)_1000_ ± SD	Total (BN_NPB_)_4000_	Distribution of BN_NPB_ Cells with	
					1 NB	2 NB					1 NPB	2 NPB
Control	2.00 ± 0.82	8	2.00 ± 0.82	8	8	0	0	0	0	0	0	0
Positive Control (Bleomycin)	14.25 ± 0.50 *	57 *	13.25 ± 0.96 *	53 *	49 *	4 c,p1,p5,p10,g1, g5,g10, p1+iri, p5+iri, p10+iri, g5+iri, g10+iri	1.75 ± 1.26 c,p1,p5,p10, g1,g5,g10, p1+iri, p5+iri, p10+iri, g1+iri, g5+iri, g10+iri	7 c,p1,p5,p10,g1,g5,g10,p1+iri, p5+iri, p10+iri, g1+iri, g5+iri, g10+iri	1.75 ± 1.26 c,p1,p5,p10, g1,g5,g10, p1+iri, p5+iri, p10+iri, g1+iri, g5+iri, g10+iri	7 c,p1,p5, p10,g1,g5,g10,p1+iri,p5+iri, p10+iri, g1+iri, g5+iri, g10+iri	7 c,p1,p5,p10,g1, g5,g10, p1+iri, p5+iri, p10+iri, g1+iri, g5+iri, g10+iri	0
Propolis 1×	2.25 ± 0.50	9	2.25 ± 0.50	9	9	0	0	0	0	0	0	0
Propolis 5×	2.75 ± 0.50	11	2.75 ± 0.50	11	11	0	0	0	0	0	0	0
Propolis 10×	3.00 ± 0.82	12	3.00 ± 0.82	12	12	0	0	0	0	0	0	0
Galangin 1×	1.75 ± 0.50	7	1.75 ± 0.50	7	7	0	0	0	0	0	0	0
Galangin 5×	2.25 ± 0.96	9	2.25 ± 0.96	9	9	0	0	0	0	0	0	0
Galangin 10×	2.25 ± 0.50	9	2.25 ± 0.50	9	9	0	0	0	0	0	0	0
Irinotecan (IRI)	11.00 ± 2.16 c,p1,p5,p10,g1,g5,g10,p1+iri, p5+iri,p10+iri, g5+iri,g10+iri	44 c,p1,p5, p10,g1,g5, g10,p1+iri, p5+iri, p10+iri, g5+iri, g10+iri	10.25 ± 1.71 c,p1,p5,p10,g1, g5,g10,p1+iri, p5+iri,p10+iri, g10+iri	41 c,p1,p5,p10, g1,g5,g10, p1+iri,p5+iri,p10+iri, g10+iri	38 c,p1,p5, p10,g1,g5, g10,p1+iri,p5+iri, p10+iri, g10+iri	3	1.50 ± 1.29	6	1.25 ± 0.96	5	4	1
Propolis 1× + IRI	4.50 ± 0.58 g1	18 g1	4.50 ± 0.58 g1	18 g1	18 g1	0	0	0	0	0	0	0
Propolis 5× + IRI	5.75 ± 1.26 c,p1,p5,p10,g1,g5,g10	23 c,p1,p5, p10,g1,g5, g10	5.75 ± 1.26 c,p1,p5,p10, g1,g5,g10	23 c,p1,p5,p10, g1,g5,g10	23 c,p1,p5, p10,g1,g5,g10	0	0	0	0	0	0	0
Propolis 10× + IRI	5.25 ± 0.96 c,p1,g1,g5,g10	21 c,p1,g1,g5,g10	5.25 ± 0.96 c,p1,p5,g1,g5,g10	21 c,p1,p5,g1, g5,g10	21 c,p1,p5, p10,g1,g5,g10	0	0	0	0	0	0	0
Galangin 1× + IRI	9.25 ± 1.50 c,p1,p5,p10,g1,g5,g10, p1+iri,p5+iri, p10+iri	37 c,p1,p5, p10,g1,g5, g10, p1+iri, p5+iri, p10+iri	8.75 ± 0.96 c,p1,p5,p10, g1,g5,g10, p1+iri,p5+iri, p10+iri	35 c,p1,p5,p10, g1,g5,g10, p1+iri,p5+iri,p10+iri	33 c,p1,p5, p10,g1,g5,g10, p1+iri, p10+iri	2	0	0	0	0	0	0
Galangin 5× + IRI	8.00 ± 1.41 c,p1,p5,p10,g1,g5,g10, p1+iri,p10+iri	32 c,p1,p5, p10,g1,g5, g10, p1+iri, p10+iri	8.00 ± 1.41 c,p1,p5,p10, g1,g5,g10, p1+iri,p10+iri	32 c,p1,p5,p10, g1,g5,g10, p1+iri, p10+iri	32 c,p1,p5, p10,g1, g5,g10, p1+iri, p10+iri	0	0	0	0	0	0	0
Galangin 10× + IRI	7.50 ± 1.29 c,p1,p5,p10,g1,g5,g10,p1+iri	30 c,p1,p5, p10,g1,g5, g10,p1+iri	7.50 ± 1.29 c,p1,p5,p10, g1,g5,g10, p1+iri	30 c,p1,p5,p10, g1,g5,g10, p1+iri	30 c,p1,p5, p10,g1,g5,g10, p1+iri	0	0	0	0	0	0	0

Microscopic evaluation was performed using a light microscope at 1000× magnification. To establish the frequencies of NBs and NPBs, the 4000 binucleated cells that contained them (i.e., BN_NB_ or BN_NPB_ cells) were scored (1000 per replicate). Data are expressed as the mean ± SD of four independent evaluations, per experimental group, and total values. The statistical significance of the results was evaluated using ANOVA with post hoc Tukey’s HSD test. The level of statistical significance was set at *p* < 0.05. The symbols and small letters indicate from which groups the relevant group differs with statistical significance: *—vs. all other experimental groups; c—vs. control; p1—vs. propolis 1× group; p5—vs. propolis 5× group; p10—vs. propolis 10× group; g1—vs. galangin 1× group; g5—vs. galangin 5× group; g10—vs. galangin 10× group; iri—vs. IRI group; p1+iri—vs. combination of propolis 1× and IRI group; p5+iri—vs. combination of propolis 5× and IRI group; p10+iri—vs. combination of propolis 10× and IRI group; g1+iri—vs. combination of galangin 1× and IRI group; g5+iri—vs. combination of galangin 5× and IRI group; g10+iri—vs. combination of galangin 10× and IRI group.

**Table 5:** Influence of apoptosis on the overall cell death observed in the experimental groups.

Experimental Group	Total No. of Nonviable Cells	Total No. of Cells in Apoptosis	Ratio
Control	16	9	0.6
Positive Control	100	59	0.6
Propolis 1×	14	8	0.6
Propolis 5×	13	7	0.5
Propolis 10×	17	8	0.5
Galangin 1×	14	7	0.5
Galangin 5×	16	8	0.5
Galangin 10×	12	8	0.7
Irinotecan (IRI)	76	42	0.6
Propolis 1× + IRI	25	14	0.6
Propolis 5× + IRI	25	16	0.6
Propolis 10× + IRI	28	17	0.6
Galangin 1× + IRI	52	28	0.5
Galangin 5× + IRI	42	23	0.5
Galangin 10× + IRI	34	19	0.6

Nonviable cells and cells in apoptosis were scored along with 4000 binucleated cells for each experimental group.

**Table 6:** Lymphocyte proliferation after treatment with propolis (at 1×, 5× and 10× concentrations), galangin (at 1×, 5× and 10× concentrations), irinotecan (IRI), combinations of propolis/galangin and IRI, and in the appropriate controls.

Experimental Group	% of Cells with 1 to 4 Nuclei				CBPI	Replication Index (%)
	M1	M2	M3	M4		
Control	14.5	72.8	5.2	7.5	1.983	100.0
Positive Control	45.5 ^↑^	46.1 ^↓^	4.2	4.2 ^↓^	1.629	64.0
Propolis 1×	12.2 ^↓^	77.5 ^↑^	3.4^↓^	6.9	1.982	99.8
Propolis 5×	11.7 ^↓^	76.2 ^↑^	4.6	7.5	2.006	102.3
Propolis 10×	14.9	73.8	4.6	6.7	1.963	98.0
Galangin 1×	13.4	74.7	4.0	7.9	1.986	100.3
Galangin 5×	13.3	76.9 ^↑^	3.3 ^↓^	6.5	1.967	98.3
Galangin 10×	10.8 ^↓^	79.0 ^↑^	4.0	6.2	1.995	101.2
Irinotecan (IRI)	43.1 ^↑^	52.9 ^↓^	2.0 ^↓^	2.0 ^↓^	1.609	62.0
Propolis 1× + IRI	16.0 ^L^	76.9 ^↑H^	3.6 ^↓H^	3.5 ^↓H^	1.912	92.8
Propolis 5× + IRI	15.6 ^L^	76.6 ^↑H^	3.5 ^↓H^	4.3 ^↓H^	1.923	93.8
Propolis 10× + IRI	15.3 ^L^	76.5 ^↑H^	3.7 ^↓H^	4.5 ^↓H^	1.930	94.6
Galangin 1× + IRI	24.5 ^↑L^	72.3 ^H^	1.4 ^↓^	1.8 ^↓^	1.788	80.1
Galangin 5× + IRI	22.6 ^↑L^	74.2 ^H^	1.6 ^↓^	1.6 ^↓^	1.806	82.0
Galangin 10× + IRI	23.1 ^↑L^	74.6 ^H^	1.0 ^↓L^	1.3 ^↓^	1.792	80.5

To establish the number of cells with 1–4 nuclei (M1–M4), slides were analysed using a light microscope at 400× magnification. Data are expressed as percentages of cells scored in four independent evaluations (4 × 500 cells), per experimental group. In line with OECD recommendations [8], the cytokinesis-block proliferation index (CBPI) and replication index (RI) were determined by using the formulae described in the Materials and Methods Section. For calculations of their values, a total of 2000 cells per experimental group was used (it represents 4 × 500 cells scored in four independent evaluations). The statistical significance of the results was evaluated using Pearson’s *χ*^2^ test (at *p* < 0.05). Arrows indicate significant increases (↑) and decreases (↓) compared to the control group. Letters indicate significant increases (H) and decreases (L) compared to the group exposed to single IRI.

## Data Availability

The data that support the findings of this study are available from the corresponding author upon reasonable request.
